# Attitudes About Brain Donation Among African American Research Participants

**DOI:** 10.1093/geroni/igaa057.2685

**Published:** 2020-12-16

**Authors:** Deborah Dyslin, Sara Dunlop, Brenda Aldridge, Robin Tillotson, Darby Morhardt

**Affiliations:** 1 Northwestern University, Chicago, Illinois, United States; 2 SouthEast (Atlas) Regional Senior Center, Chicago, Illinois, United States; 3 Chicago Department of Family & Support Services, Chicago, Illinois, United States

## Abstract

Alzheimer’s and related dementias (ADRD) disproportionately affect the African American community. Brain donation, a crucial part of translational research, is less common among African American research participants compared to White research participants at Alzheimer’s Disease Research Centers (ADRCs) across the US. Existing literature suggests three categories of contributory factors for African Americans: concerns and misconceptions about brain research and brain donation; religious beliefs; and the role of the family. Existing knowledge of community interventions is limited. We conducted seven focus groups, stratified by brain donation intent and cognitive status, to capture the perspectives of African American research participants. Qualitative content analysis reveal the following contributory themes: personal connection to memory loss or dementia; altruism; spirituality/religion; historical and current racism in health care and research; trauma and objectification; trust; representation; understanding the purpose and process of brain donation; and fluidity in decision-making. Future research will explore trauma-informed and culturally responsive interventions. Part of a symposium sponsored by the Alzheimer’s Disease Research Interest Group.

